# Evaluation of dynamic change in regenerated tendons in a mouse model

**DOI:** 10.1186/s40634-018-0152-6

**Published:** 2018-09-21

**Authors:** Yoshinori Ohashi, Junsuke Nakase, Kengo Shimozaki, Kojun Torigoe, Hiroyuki Tsuchiya

**Affiliations:** 1 Department of Orthopaedic Surgery, Graduate School of Medical Sciences, Kanazawa University, Kanazawa, Japan; 2 Department of Rehabilitation, Fukui Health Science University Faculty of Health Science, Fukui, Japan

**Keywords:** Tendon gel, Tendon regeneration, Film model method, Intrinsic healing, Traction force

## Abstract

**Background:**

Using the film model method, the process whereby a substance called tendon gel is secreted from transected tendon ends and changed into a tendon after application of a traction force is known. The objective of this study was to investigate the association between mechanical properties in the early stages of tendon regeneration and time by using the film model method.

**Method:**

Adult male ddY mice, closed colony mice established and maintained in Japan, were prepared for each experimental group. The study animals were 30 mice and were divided into three groups of 10 mice each. Ten specimens of tendon gel secreted from the transected tendon ends were collected on days 10, 15, and 20 postoperatively. While a traction force of 0.00245 N was applied to these specimens, the process of tendon gel changing into a tendon was video recorded for 24 h, and the length of extension was measured over time. Regenerated tendons were stained with hematoxylin and eosin for histological examination. Healing site was studied histologically according to the our maturity score with reference to the Bonar’s scale.

**Results:**

The day 10 specimens gradually stretched for 12 h after the start of pulling and transformed into tendons. In contrast, the day 15 and 20 specimens stretched immediately after the start of pulling and transformed into tendons. The day 10 specimens stretched significantly more than the day 15 and 20 specimens (mechanical strain; 0.43 ± 0.26%, 0.03 ± 0.02%, and 0.03 ± 0.01%, respectively)Statistically significant differences were observed in the day 10 specimens than in the day 15 and 20 specimens. (*P* < 0.017). Using our maturity scores, the day 15 and 20 specimens were more mature than the day 10 specimens. (1.6 ± 0.68, 3.9 ± 0.54, and 4.8 ± 0.64, respectively) Statistically significant differences were observed in the day 10 specimens than in the day 15 and 20 specimens (*P* < 0.017).

**Conclusion:**

Tendon gel physiologically and histologically matures on or after day 15 and becomes stronger dynamically in mechanical strength after day 15 than after day 10.

## Background

The injured tendon healing process consist of the following phases: inflammatory, proliferative, and remodeling. Many researchers have been studying various factors, such as growth factors and stem cells, and treatments to reduce inflammation and to investigate this process. They demonstrated that there are no perfect factors to shorten the healing process without encountering problems of adhesion and mechanical strength (Awad et al., 2003; Muller et al., 2016; Güleç et al., 2018). Moreover, other healing processes include intrinsic and extrinsic healing. It has been considered that tendons have limited intrinsic healing capacity (Torigoe et al., 2011; Manske and Lesker, 1984). However, previous studies demonstrated that tendons have both extrinsic and intrinsic healing capacities (Manske and Lesker, 1984; Mass and Tuel, 1989; Mass and Tuel, 1990; Brik et al., 1990). In general, tendons regenerated during the extrinsic and intrinsic healing processes are inflexible. Neovessels invading the surrounding tissue during the extrinsic healing process and adhesions to tendon sheaths and other surrounding tissue lead to inflexibility of the regenerated tendon. Regenerated tendon strength is weak, because the injured tendon develops from a scar tissue (Ahmed et al., 1998). Thus, gliding capacity and mechanical strength are lower after healing compared to those before injury. It has not been elucidated when and how tropocollagen secreted by tenocytes aggregating at the injury site in the repair phase converts to collagen fibrils and becomes aligned (Goulet et al., 1997).

Given the technical difficulty in clearly distinguishing between the intrinsic and extrinsic tendon healing processes in vivo, the details of either process have not been elucidated. Torigoe et al. (2011) reported that they were able to partially observe the intrinsic tendon healing process by placing the ends of transected mouse Achilles tendons between two sheets of thin film, which is called the film model method. They used the film model method to observe intrinsic tendon healing; this method was originally used for the regeneration of peripheral nerves (Torigoe et al., 1999). Given the very narrow space between the two shielding films covering the cut end of the tendon, only body fluids could pass through the films, as they separated the cut end of the tendon from the surrounding tissue (Torigoe et al., 2011). Using this method, Torigoe et al. (2011) also reported that the extracellular matrix is secreted from tendon stamps and spread onto the film. This extracellular matrix with gel-like consistency was called a tendon gel, which transformed into tendons by applying a traction force on or after day 11. Their report appears to partially describe the intrinsic tendon healing process, which currently remains unknown.

### Hypothesis

We thought that we could partially elucidate this process further through dynamic and continuous observation of tendon healing using a light microscope. We performed video recordings using the stereomicroscope and recorded changes in the 24-h elongation rate. Torigoe et al. (2011) observed only the static change after the traction. Sasaki et al. (2012), who observed healing of mouse Achilles tendons through scanning electron microscopy, reported that randomly aligned fibers appeared on day 7 of the observation and were aligned in one direction by day 14. Güngörmüş and Kolankaya (2012), who measured the weekly expression of the *Scx* gene, a marker for tenocytes, reported that gene expression peaked in the second week after injury. On the basis of these reports, we proposed a hypothesis that secretion of tropocollagen from tenocytes migrating from transected tendon ends and formation of collagen fibers from tropocollagen in the healing process peak on or after day 15 after the injury, rather than on day 10 as reported by Torigoe et al. (2011). Using an electron microscope, Torigoe et al. (2011) reported that the tendon gel did not have a layered structure on day 10 and only had a layered structure on day 13. We wanted to investigate the change before and after the layered structure was formed; hence day 10 was used as the start point.

### Aim

The objective of this study was to investigate the peak period of the production of collagen fibers through a dynamic observation of the tendon gel to which a traction force is applied continually for 24 h combined with a histological examination of the resulting tendon gel.

## Methods

**Fig. 1:**
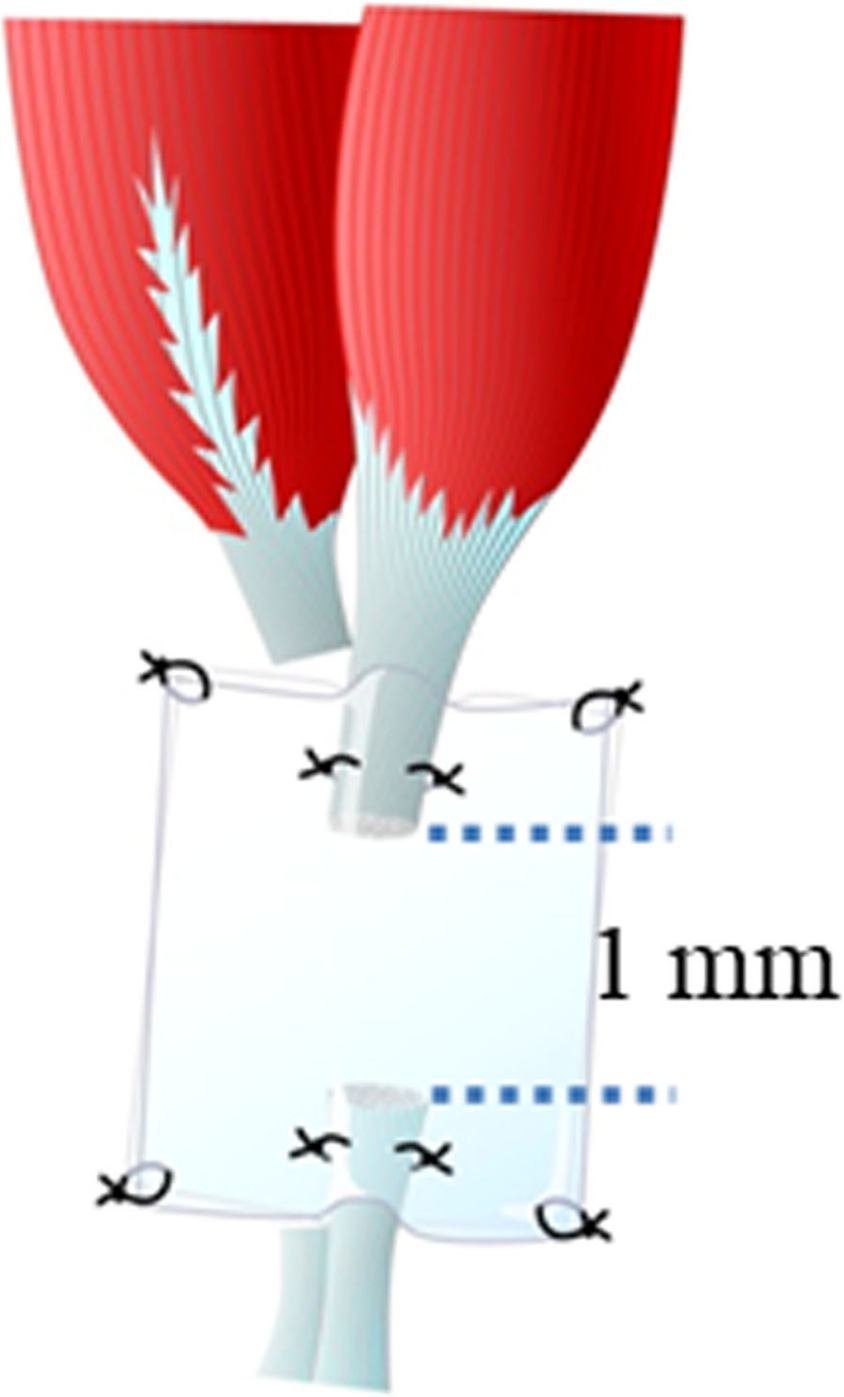
Scheme of the film model method. Proximal and distal ends of the transected tendon are placed on a thin plastic film. The two cut ends face each other and are separated by approximately 1 mm

**Fig. 2:**
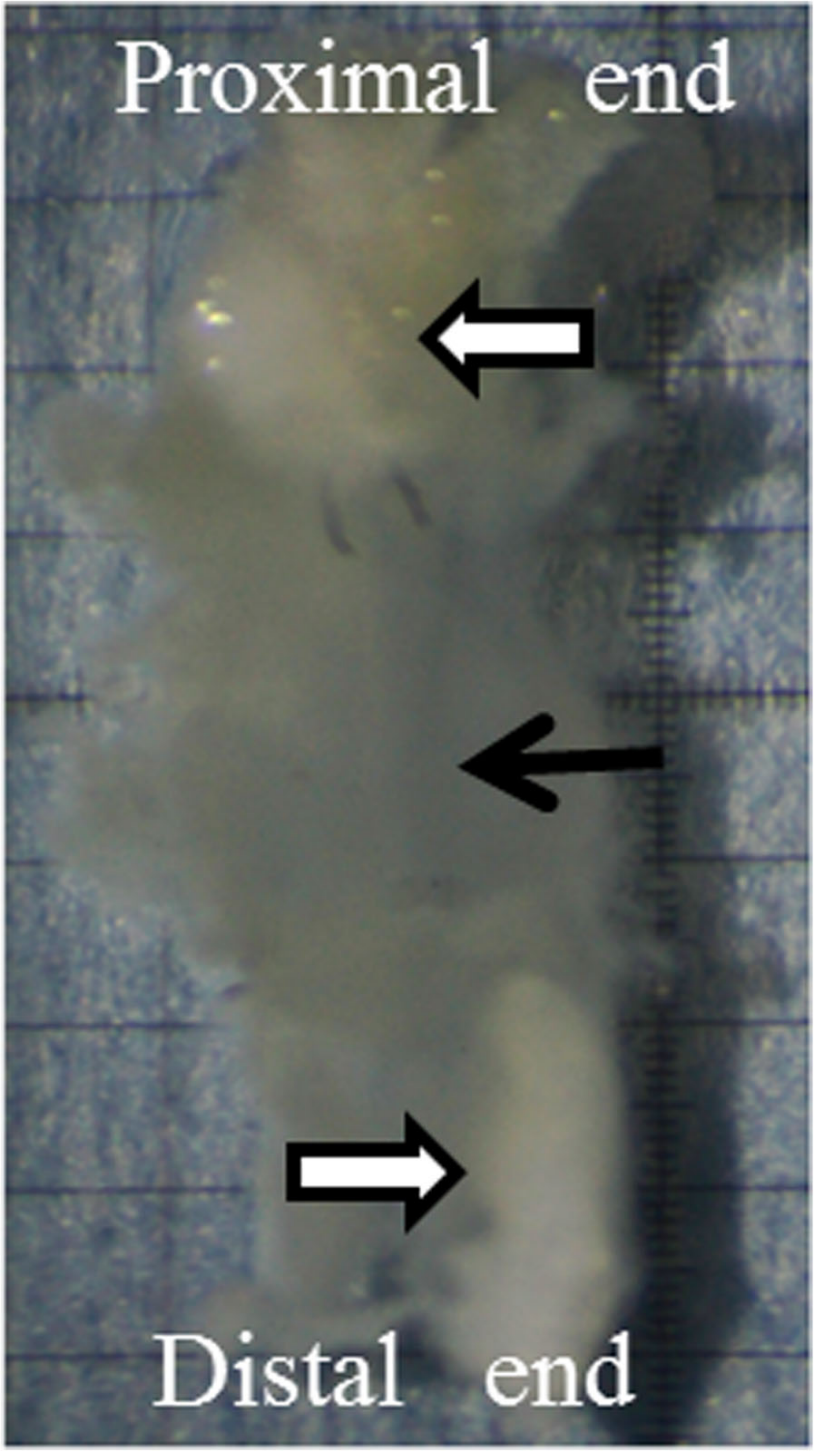
Stereomicroscopic images of the tendon gel. The tendon gel (black arrow) with tendon stamps (white arrow) is shown

This experiment was carried out as a controlled laboratory study. All animals used for the animal experiments were approved by the Animal Experimental and Use Committee of the Institute for Experimental Animals, Kanazawa University Advanced Science Research Center. The experiments were performed according to the animal experiment regulations of the institute. Adult male ddY mice, closed colony mice established and maintained in Japan, were purchased from Japan SLC (Shizuoka, Japan). The study animals were 30 mice weighing 20 to 30 g, and they were divided into three groups of 10 mice each. For surgery, the mice were anesthetized by inhalation of 1 mL of isoflurane in a 200-mL glass chamber. While a 20-mL syringe jacket containing absorbent cotton was used as a nose cone, the concentration of anesthetic in the jacket was one-half of that in the glass chamber. In the film model method, an area ranging from the left gastrocnemius muscle to the Achilles tendon enthesis was first exposed, and the medial head of the gastrocnemius muscle was then transected. The proximal and distal ends of the transected tendon were placed opposite each other and separated by 1 mm on a sheet of thin fluorine resin film (25-μm thick, 3 × 5 mm, Aflex 25 N NT, Asahi Glass, Tokyo, Japan) and fixed with a 10–0 nylon thread (Keisei Medical Industrial Co., Ltd., Tokyo, Japan). After a few drops of Ringer solution (Fuso Pharmaceutical Industries, Osaka, Japan) were applied to moisten the ends, they were covered and fixed with another sheet of the film (Fig. 1). The four corners of the films were also fixed using a 10–0 nylon thread. If necessary, some sutures with 10–0 nylon thread were added at the edge of the films. After surgery, the mice were administered 2 ml of 2% acetaminophen (AYUMI Pharmaceutical Corporation, Tokyo, Japan) orally. These ends were left in the body for 10 days (day 10 group: D10), 15 days (day 15 group: D15), or 20 days (day 20 group: D20) after the tendon surgery. After the mice were euthanized with isoflurane on day 10, 15, or 20, the tendon gel together with the transected tendon ends was collected (Fig. 2).

### Stereomicroscopic kymography

While the collected specimens were fixed at one end under moist conditions, the other end was pulled at a force of 0.00245 N for 24 h. We dropped a Ringer ‘s solution onto the tendon gel for 24 h so that the tendon gel was always filled with the Ringer’ s solution. We placed the specimens in an area away from the sunlight with normal temperature and pressure. The traction force was determined according the report of Torigoe et al. (2011). They reported that the traction force was the maximum tension that could be added without breaking the tendon gel on day 10. This process was video recorded using the stereomicroscope for 24 h with a micro ruler. The extended length of the tendon gel was measured after the start of pulling at 5, 10, and 30 min and at 1, 2, 12, and 24 h. The length was compared among the D10, D15, and D20 groups in these videos. Applying traction on the tendon gel was performed in vitro.

### Electron microscopy

After 24 h of applying traction, the specimens were immersed in Karnovsky’s fixative, and then in 2% osmium tetroxide, and dehydrated using ethanol series. After removing the underlying film, the specimens were embedded in Epon 812. Ultrathin sections obtained by using a diamond knife were stained with uranyl acetate and lead citrate for Transmission Electron Microscope (TEM) (JEM 1200EX II; JEOL, Tokyo, Japan). The central part of the tendon gel was observed.

### Histological examination

After 24 h of applying traction, the tendon gel specimens were stained with hematoxylin and eosin. Torigoe et al. (2011) reported that round cells migrated from the tendon stump to the tendon gel, flat cells appeared around the lamellar tendon gel, and the application of mechanical stress induced a transformation of the lamellar tendon gel into the aligned collagen fibrils. On the basis of this report, the tendon gel that transformed into collagen fibers by traction force was termed matured tendon gel. Evaluation was performed by semi-quantifying the maturity of the tendon gel. We considered that scoring was necessary to observe the maturity of the tendon gel. The scale commonly used for histological evaluation of tendons is the Bonar’s histological scale, which was validated by Maffulli et al. (Cook J et al., 2004, Maffulli et al., 2008). On the other hand, in assessing the maturity of the tendon gel, it was considered that maturity differs between the tendon-gel junction and the central part of the gel, so we created a maturity score with reference to the Bonar’s histological scale. The following parameters of the tendon gel specimens were examined with a light microscope (KEYENCE BZ-900, Keyence, Osaka, Japan) of 200 times power: (1) the ratio of the cell count in the tendon-gel junction (Tgj) to that in the central part of the gel (C), (2) presence or absence of collagen fibers in C, (3) presence or absence of round cells in C, (4) ratio of round cells to flat cells in C, and (5) vascularity within the tendon gel.

These parameters were converted into numerical scores and used as indices of tendon maturity (Table 1). The observation sites were randomly selected from both the Tgj and C of the gel. Two examiners independently assessed the specimens for those parameters, and the mean values of scores determined by both examiners were used as the maturity scores.

**Table 1:** Maturity scores of the tendon gel

	Score 0	Score 1	Score 2
Cell count ratio (Tgj/C)	> 1	≤1	
Collagen fibers	Absent	The loss of demarcation	The separation of individual fibers
Round cells	Absent	Increased roundness	Amount of cytoplasm visible
Ratio of flat cells to round cells	0	< 1	≥1
Vascularity	Absent	Presence	

*Tgj* tendon gel junction, *C* the central part of the tendon gel

Two observation points (Tgj and C) are set. The presence and absence of fibers and the number of cells are counted under a light microscope. The score is evaluated by semi-quantifying the maturity of the tendon gel

### Statistical analysis

Quantitative data were expressed as mean ± standard deviation (*n* = 10). Shapiro-Wilk normality test was performed on the obtained values, with significance level set at *P* > 0.05. Intergroup comparison before and after tendon pulling was performed by using one-way ANOVA with post-hoc Tukey test. A critical rate (*P* value) when using the Bonferroni correction (*p*  <  0.05/3  =  0.017) for multiple comparisons was considered significant. Data were analyzed using SPSS for Windows 23.0 (IBM Corp., Armonk, NY, USA).

## Results

### Stereomicroscopic kymography

**Fig. 3:**
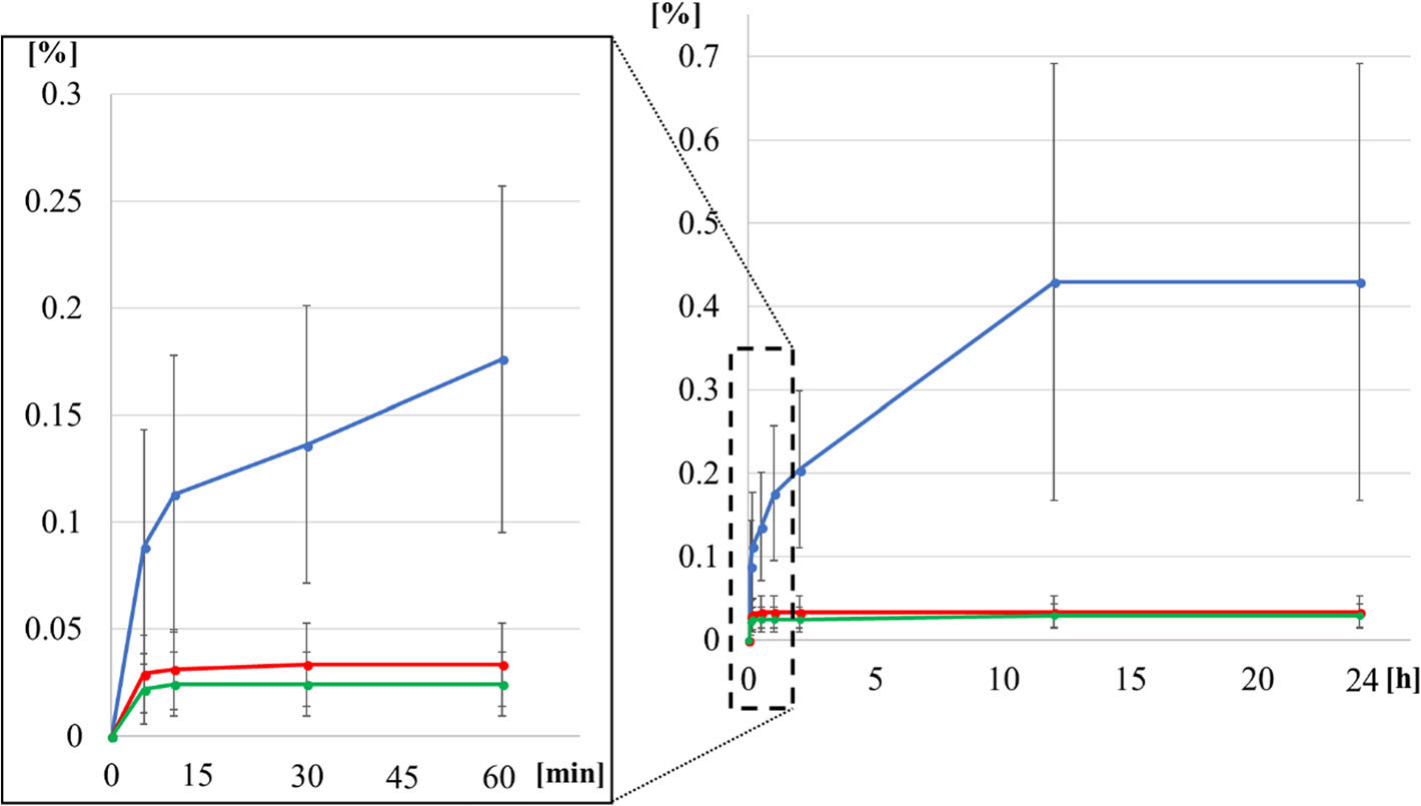
Mechanical strain (ε) of the tendon gels. Mechanical strain (ε) of the tendon gels over time on days 10, 15, and 20. On day 10, mechanical strain (ε) is stopped 12 h later. On days 15 and 20, the mechanical strain (ε) is stopped only 5 min later

**Fig. 4:**
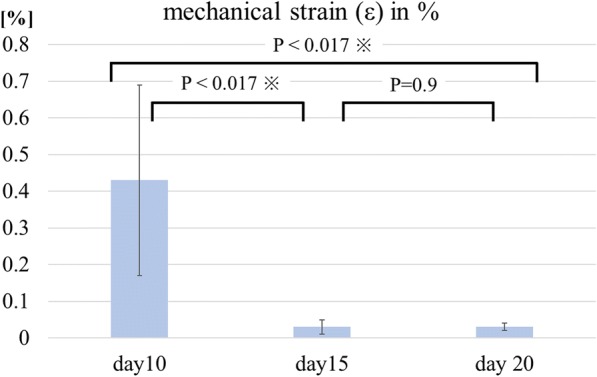
Graph of mechanical strain (ε) of the tendon gel after applying traction force for 24 h. mechanical strain (ε) of tendon extension after applying traction on days 10, 15, and 20. mechanical strain (ε) of the tendon gels are significantly different between the day 10 and 15 groups and the day 10 and 20 groups

After 24 h of pulling, the tendon gel was stretched. When expressed using a mechanical strain (ε), it was 0.43 ± 0.26% in D10, 0.03 ± 0.02% in D15, and 0.03 ± 0.01% in D20. The mechanical strain in D10 was significantly higher than that in D15 and D20 (*P* < 0.01); however, no significant difference was observed between D15 and D20 (*P* = 0.9) (Figs. 3 and 4). In D10, one-third of the total extension occurred in the first hour after the start of traction; however, the specimens were not stretched after 12 h. In D15 and D20, total extension was completed within 10 min after the start of traction.

### Electron microscopy

**Fig. 5:**
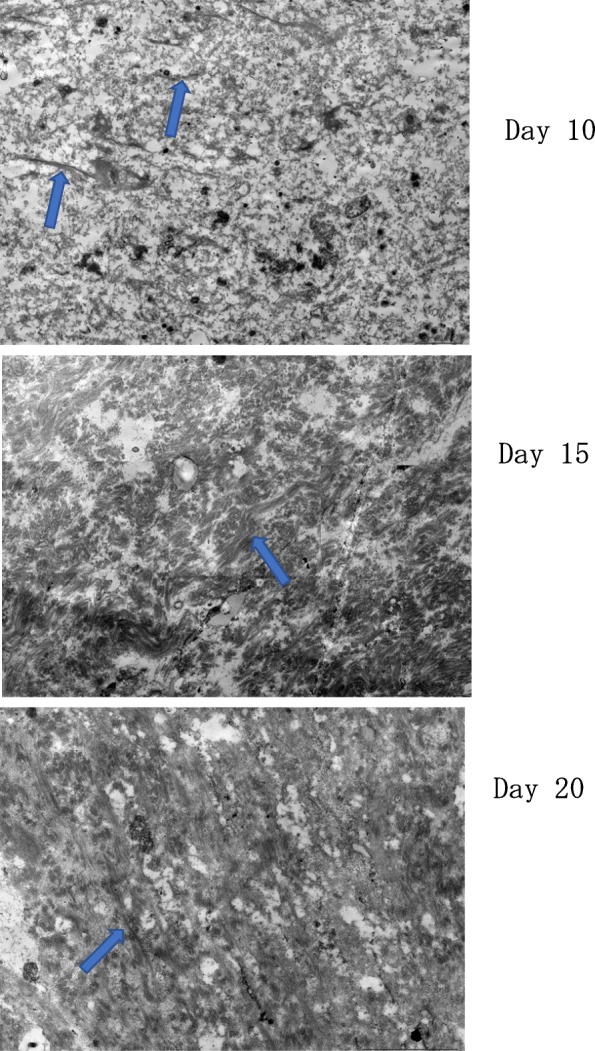
Observation of tendon gel specimens using a transmission electron microscope. On day 10, the collagen fibers are thin and loose. On day 15, the collagen fiber becomes clear and denser. On the day 20, a stripe pattern peculiar to the collagen fibers are noted. Arrow: collagen fibers; scale bar: 2 μm

On day 10, thin collagen fibers were sparsely distributed and loosen in the tendon gel. On day 15, the collagen fiber became clear and denser than on day 10. On day 20, a stripe pattern peculiar to the collagen fibers, which could not be confirmed on day 15, was observed (Fig. 5). New blood vessels were not observed in each specimen.

### Histological examination

**Fig. 6:**
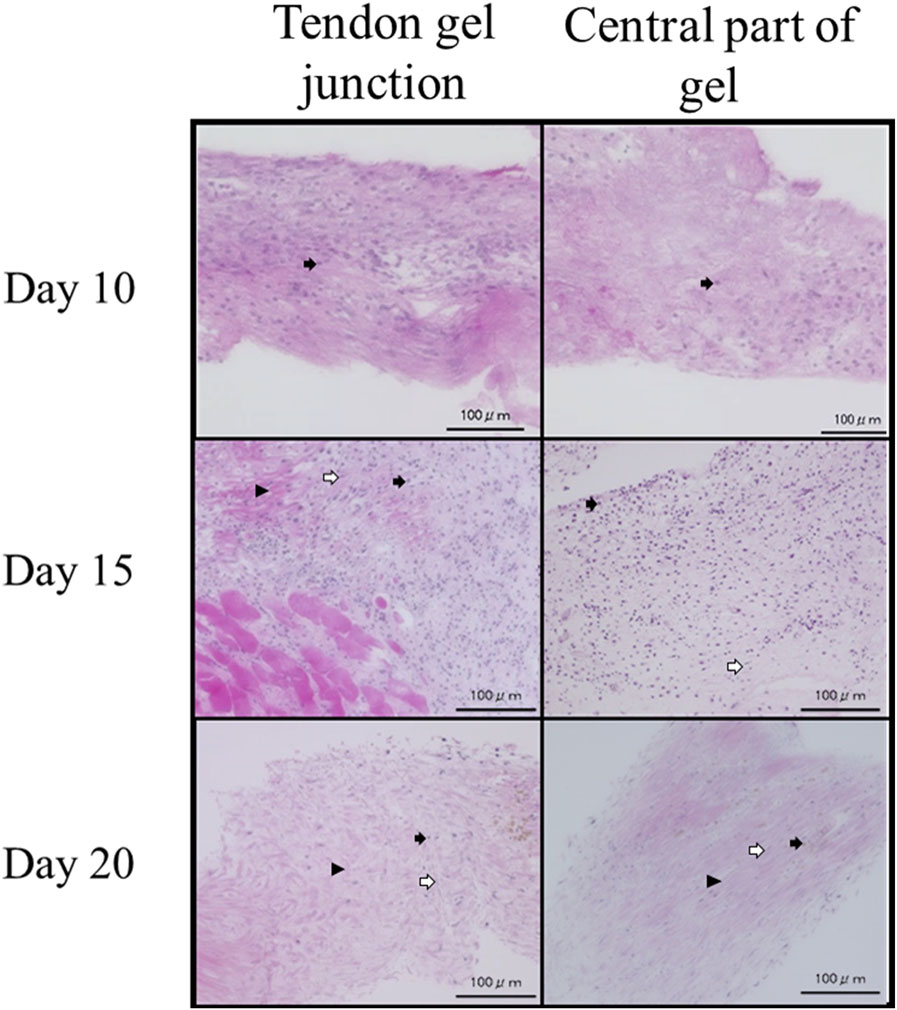
Observation of tendon gel specimens stained with hematoxylin and eosin under a light microscope. Round cells, flat cells, and collagen fibers are observed. Collagen fibers are not observed in the day 10 group. Round cells are decreased at the central part of the tendon gel in the day 20 group than in the day 15 group. Arrowhead: collagen fibers, white arrow: round cells, black arrow: flat cells

**Fig. 7:**
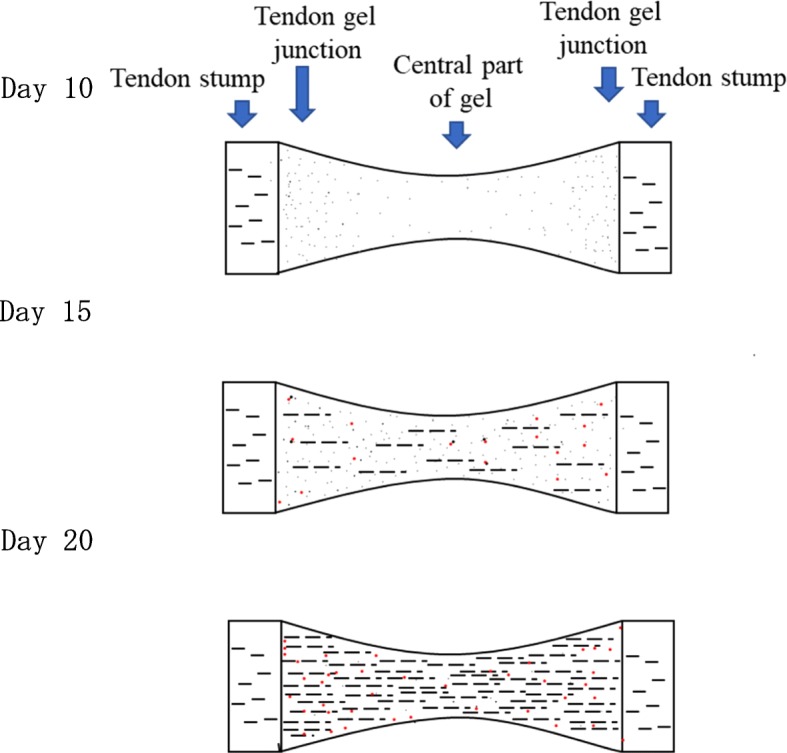
Scheme of cell and fiber distribution changes. The trend of the local existence of cells and fibers. Black dot: round cells, red dot: flat cells, black line: collagen fibers

In D10, round cells were extensively present in Tgj, and collagen fibers were observed around the tendon stump. On the other hand, in the C region, although round cell migration was observed, there was an area without round cells. Some specimens had demarcation losses of collagen fibers. In the histology of D15 specimens, the amount of round cells was increased in both Tgj and C regions. A small amount of flat cells could be observed in either area. The separation of individual collagen fibers appeared in any specimen at Tgj, but there were many collagen fibers not clearly defined in the C region. In most D20 specimens, the collagen fibers could be clearly identified. Although the amount of flat cells had increased, the amount of round cells was even more. Neovascularization was not observed in any group. Moreover, collagen fibers were not observed in D10 but were detected in D15 and D20 (Figs. 6 and 7). The maturity scores were 1.6 ± 0.68 in D10, 3.9 ± 0.54 in D15, and 4.8 ± 0.64 in D20. The score in D10 was significantly lower than the scores in D15 and D20 (*P* < 0.01); however, no significant difference was observed between D15 and D20 (*P* = 0.04; using the Bonferroni correction, *P*  <  0.017) (Tables 2 and 3).

**Table 2:** Summary of the maturity scores between the groups

Maturity score	Day 10	Day 15	Day 20
	(*N* = 10)	(*N* = 10)	(*N* = 10)
Mean	1.6	3.9*	4.8*^#^
SD	0.68	0.54	0.64
Range	0–3	3–4	4–6

^*^P < 0.001, vs. day 10; ^#^*P* = 0.04, vs. day 15

*P* value when using the Bonferroni correction (*p*  <  0.05/3  =  0.017)

The score in the D10 group is significantly lower than the scores in the D15 and D20 groups; however, no significant difference is observed between the D 15 and D20 groups

**Table 3:** Distribution of the tendon gel maturity score

Variable	Day 10 (*N* = 10)			Day 15 (*N* = 10)			Day 20 (*N* = 10)		
	0	1	2	0	1	2	0	1	2
① Cell count ratio (Tgj/C)	8	2		3	7		0	10	
② Collagen fibers in C	5	5	0	0	8	2	0	4	6
③ Round cells in C	1	9	0	0	10	0	0	8	2
④ Ratio of flat cells to round cells in C (F/R)	10	0	0	0	10	0	0	10	0
⑤ Vascularity	10	0		10	0		10	0	

## Discussion

In this study, angiogenesis did not occur with the film model method. A tendon gel is secreted only at the transition of cells from the tendon stump, and we consider this as an intrinsic healing process. Thus, we considered that this study evaluated the intrinsic healing of mouse Achilles tendons on days 10, 15, and 20 after surgery using the film model method. The results demonstrated that the application of a traction force to the tendon gel secreted from the transected tendon ends induces tendon regeneration on day 10, and that application of a traction force on or after day 15 results in the regeneration of the tendon that is more resilient to a traction force.

Kuzumaki et al. (2017) reported that a strong traction force applied to the tendon gel can form thick fibers. Considering these reports, it is thought that the thick fibers are more resistant to tension, and there is a possibility that the results in D15 and D20 would change if a strong force within the range that does not break the tendon gel is applied in D15 and D20. In D10, extension stopped 12 h after the application of a traction force of 24.5 × 10^− 4^ N. In contrast, extension stopped within 5 min after the application of a traction force in D15 and D20. We assumed that extension stops when the tendon gel becomes resilient to a traction force. While it is generally considered that the physical stability of collagen fibers is more affected by intermolecular cross-links than by intramolecular cross-links, collagen fibers are more strongly bound together when more fibers are cross-linked than when collagen molecules are larger (Graham et al., 1984; Klein et al., 2001). Moreover, Kuhn (1987) reported that the cross-linking of collagen fibrils lends tensile strength to tendons, whereas Kwansa et al. (2014) indicated that the application of a traction force promotes cross-linking between the amino and carboxy I terminals of the collagen fibers, which structurally strengthen the collagen tissue. Furthermore, mechanical stress is essential for the maturity of collagen fibers (Urschel et al., 1988). Similarly, Kuzumaki et al. (2017) reported that when the cross-linking reaction progressed, collagen bundles were formed by a Schiff-base cross-linking reaction, and the bundle became thicker over time.

Based on these reports, the cross-link between collagen fibers in a regenerated tendon appears to be weaker on day 10 than on days 15 and 20 after surgery, because the tendon gel in D10 was stretched more by pulling than that in the other groups. Thus, it is assumed that tendon gel in D10 might have contained a small amount of collagen molecules, which might have resulted in weak cross-linking (Graham et al., 1984; Klein et al., 2001). When using an electron microscope, we observed that collagen fibers became clear and denser on day 15, and that a stripe pattern peculiar to the collagen fibers also appeared on day 20. In other words, we consider that collagen molecules may have increased in the tendon gel during the first 15 days after transection and become more likely to form intermolecular cross-links, which resulted in mature tendon gel (i.e., likely to become tendon). It is considered that collagen fibers mature in a two-step reaction consisting of (1) nucleation by crystal growth, which is well known in common inorganic ions and low-molecular substances, and (2) secondary growth caused by the alignment of fibers in one direction. We believe that the gradual increase in tensile strength in this study can be explained by nucleation and the secondary growth of nuclei. Tendons generally contain only a few flat tenocytes. Extracellular matrix and water compose 70% of a tendon, while the collagen fibers compose the remaining 30% (Evans and Barbenel, 1975; Butler et al., 1978). In this study, many round cells were found at the tendon injury sites. When a tendon is injured, tenocytes migrate to the injury site, where they synthesize and secrete tropocollagen molecules (Prockop and Kivirikko, 1995; Sharma and Maffulli, 2005). Then, the tropocollagen molecules lose N- and C-terminal propeptides and become collagen molecules. The collagen molecules are polymerized and cross-linked to become collagen fibrils, which aggregate to form collagen fibers (Graham et al., 1984; Klein et al., 2001; Prockop and Kivirikko, 1995).

We quantified and assessed the degree of maturity based on these findings. Although the tendon gel specimens in D15 and D20 were more mature than those in D10, no difference was observed between D15 and D20. Thus, the appropriate timing of applying a traction force after injured tendons healed seemed to be on or after day 15 after injury.

This study has certain limitations. First, as the applied pulling force was fixed to the traction force that was the maximum tension that could be added without breaking the tendon gel on day 10, it cannot be denied that the tendon gel specimens in D15 and D20 might have been resilient to a greater traction force. There is a possibility that the tensile strength will differ between D15 and D20. Then, the mechanical strain will change, but it is unclear whether there is no difference between the values of D15 and D20. Second, the validity of the semiquantified degree of maturity was not examined at the cellular level. Third, we did not evaluate the specimens before traction. Fourth, the size of collagen fibers was not directly assessed through electron microscopy or other methods. Fifth, we did not perform an immune histology. Thus, we suggest that future investigators should include an immune histology in their research. Finally, there are experiments which are presumed from the previous papers but have not been confirmed in our study; hence, these should be investigated in future studies as well.

## Conclusions

Tendon gel was mechanically and histologically more mature on day 15 than on day 10 after surgery, and the degree of maturity appeared comparable between tendon gel on days 15 and 20.

## Abbreviations

C: the central part of the gel
D10: day 10 group
D15: day 15 group
D20: day 20 group
Nm: nanometer
Tgj: tendon gel junction
